# *SIRT1*, miR-132 and miR-212 link human longevity to Alzheimer’s Disease

**DOI:** 10.1038/s41598-018-26547-6

**Published:** 2018-05-31

**Authors:** A. Hadar, E. Milanesi, M. Walczak, M. Puzianowska-Kuźnicka, J. Kuźnicki, A. Squassina, P. Niola, C. Chillotti, J. Attems, I. Gozes, D. Gurwitz

**Affiliations:** 1 Department of Human Molecular Genetics and Biochemistry, Sackler Faculty of Medicine, Tel Aviv University, Tel Aviv, 69978 Israel; 2 Department of Cellular and Molecular Medicine, Victor Babes National Institute of Pathology, Bucharest, Romania; 3 Institute of Genetics and Animal Breeding, Polish Academy of Sciences, Warsaw, Poland; 4 Department of Human Epigenetics, Mossakowski Medical Research Centre, Warsaw, Poland; 5 Department of Geriatrics and Gerontology, Medical Centre of Postgraduate Education, Warsaw, Poland; 6 The International Institute of Molecular and Cell Biology, Warsaw, Poland; 7 Department of Biomedical Sciences, University of Cagliari, Cagliari, Italy; 8 Unit of Clinical Pharmacology, University Hospital of Cagliari, Cagliari, Italy; 9 Institute of Neuroscience and Newcastle University Institute of Ageing, Newcastle University, Newcastle upon Tyne, UK; 10 Adams Super Center for Brain Studies, Sagol School of Neuroscience, Tel Aviv University, Tel Aviv, Israel

## Abstract

Alzheimer’s Disease (AD) is the most common cause of dementia in the elderly. Centenarians – reaching the age of >100 years while maintaining good cognitive skills – seemingly have unique biological features allowing healthy aging and protection from dementia. Here, we studied the expression of *SIRT1* along with miR-132 and miR-212, two microRNAs known to regulate *SIRT1*, in lymphoblastoid cell lines (LCLs) from 45 healthy donors aged 21 to 105 years and 24 AD patients, and in postmortem olfactory bulb and hippocampus tissues from 14 AD patients and 20 age-matched non-demented individuals. We observed 4.0-fold (P = 0.001) lower expression of *SIRT1*, and correspondingly higher expression of miR-132 (1.7-fold; P = 0.014) and miR-212 (2.1-fold; P = 0.036), in LCLs from AD patients compared with age-matched healthy controls. Additionally, *SIRT1* expression was 2.2-fold (P = 0.001) higher in centenarian LCLs compared with LCLs from individuals aged 56–82 years; while centenarian LCLs miR-132 and miR-212 indicated 7.6-fold and 4.1-fold lower expression, respectively. Correlations of *SIRT1*, miR-132 and miR-212 expression with cognitive scores were observed for AD patient-derived LCLs and postmortem AD olfactory bulb and hippocampus tissues, suggesting that higher *SIRT1* expression, possibly mediated by lower miR-132 and miR-212, may protect aged individuals from dementia and is reflected in their peripheral tissues.

## Introduction

With increased longevity in recent decades, humankind is faced with a continued increase in the prevalence of late-onset neurodegenerative disorders, most notably, sporadic Alzheimer’s Disease (AD). In 2012 The World Health Organization has estimated that by 2050, the number of AD patients globally will triple from 35 million to 115 million. Such scenario, supported by recent data^1^, will dramatically affect healthcare-related costs and the economy, in particular in developed countries where fertility rates are declining^2^. At the same time, higher numbers of individuals are expected to reach the age of 100 years without dementia^3^. While extensive research efforts have been allocated to studying AD complex etiology and pathophysiology, including large epidemiological studies^4^, genome-wide association studies^5^, and transcriptomic studies^6^, little efforts, in comparison, have been directed toward studying the genomics and epigenomics of healthy aging, which may provide clues for understanding the molecular basis for neurodegeneration and its protective factors^7^. Understanding the biology of healthy human aging and dementia-free longevity is key for deciphering the biology of neurodegenerative diseases^8^. Indeed, centenarians, individuals reaching the age of over 100 years while retaining good cognitive skills, have been extensively studied^9–11^. For example, centenarian LCLs were shown to possess enhanced DNA repair capacity following H_2_O_2_ exposure^12^.

AD frequently results, already in its early phases, in impaired olfactory sensing capacity and neuropathological hallmark lesions of neurodegenerative diseases are present in the olfactory bulb, often at early, subclinical stages^13^ Olfactory deficits can be noticed in the ability to detect, recognize, and remember odorants^14^. Olfactory impairment is associated with incident amnestic MCI and progression from amnestic MCI to AD dementia^15^. Relative proteome measurements in postmortem olfactory bulb tissue from AD have discovered protein networks interaction during the progression of sporadic AD^16^. Neurogenesis during adult life is crucial for maintaining correct synaptic connectivity and involves the generation of new axons and dendrites. Adult neurogenesis in humans takes place exclusively in the dentate gyrus of the hippocampus and in the subventricular zone of the olfactory bulb, while newborn neurons migrate to other brain regions. Impaired adult neurogenesis, implicated in AD, is underlined by reduced ability for neural stem cell renewal in these brain tissues^17^.

In our recent work^18^, we reported reduced expression of *SIRT1* (Sirtuin 1) and *RGS2* (regulator of G-protein signaling 2) in lymphoblastoid cell lines (LCLs) from AD patients compared with healthy controls. SIRT1 (silent information regulator 1) is one of seven human sirtuins that possess mono-ADP-ribosyltransferase or deacylase activity toward target proteins, including histones. SIRT1 regulates endocrine, mitochondrial, circadian rhythm and hypothalamic functions. In the brain, SIRT1 takes part in memory formation by modulating synaptic plasticity, promoting axonal elongation and dendritic branching^19^. Knock-in induced higher expression of the *Caenorhabditis elegans* and yeast homolog of human *SIRT1*, Sir2, was found to increase their lifespan^20,21^. Transgenic mice overexpressing brain *Sirt1* exhibit a delayed aging phenotype and extension of lifespan^22^. Injection of SIRT1 lentivirus in the hippocampus of p25 transgenic mice, a model of AD and tauopathies, protected the mice against neurodegeneration^23^. SIRT1 had lower concentration in serum of AD and MCI patients compared with healthy individuals^24^. *SIRT1* expression in Tg2576 mice and in derived primary neuronal cultures promotes α-secretase activity with reduction of Aβ generation, suggesting that SIRT1 prevents amyloid neuropathology^25^. During embryogenesis of mice, Sirt1 interacts with Bcl6 to promote proper neurogenesis of the mouse cerebral cortex^26^. Sirt1 was upregulated in mouse neural progenitor cells by oxidative stress and directed these cells to differentiate to astrocytes instead of neurons^27^. Interestingly, there is an association between SIRT1 activation and inhibition of Aβ generation: inhibiting SIRT1 through virally mediated expression of a dominant negative SIRT1 construct increased the accumulation of Aβ peptides by Tg2576 neuron cultures^28^. Exploring the blood gene expression profiles of centenarians along with neurodegenerative disease patients may yield important clues into longevity and protection from neurodegeneration. Among genes associated with healthy aging, those coding for sirtuins, NAD^+^-dependent protein deacylases that cleave off acetyl or other acyl groups from the ε-amino group of lysine residues in histones and other proteins, have received extra attention, as their high expression levels were demonstrated to contribute to longevity and to the lifespan-prolonging effects of caloric restriction in diverse organisms including yeast and mammals^29,30^. Among the seven mammalian sirtuins, sirtuin 1, encoded in humans by *SIRT1*, is best recognized as associated with longevity and neuroprotection^31,32^.

miRNAs, short noncoding RNA regulators of gene expression, are expressed in the brain and participate in regulating neuronal plasticity, function and development. Dysfunction in miRNA transcription may lead to neurodegenerative disease and neurodevelopment disorders^33^. For example, miR-132 contributes to dendritic outgrowth of newborn neurons in the adult mouse hippocampus^34^. miR-132, miR-212 and miR-22 were shown to target *SIRT1*^35–37^. Deletion of miR-132 and miR-212 was shown to induce tau aggregation in mice expressing endogenous or human mutant tau^38^, and impair mouse cognitive skills^39^. Additionally, these miRNAs were upregulated in postmortem frontal cortex tissues of AD and mild cognitive impairment (MCI) patients^40^.

In the current study, we therefore studied *SIRT1*, *RGS2*, miR-132, miR-212 and miR-22 expression levels in LCLs from healthy donors of various age groups, including centenarians, and in LCLs from AD patients. Next, we compared the differential expression levels of the above genes in postmortem olfactory bulb and hippocampal tissues from AD patients and non-demented age matched controls. Our findings provide insights into AD etiology, neurodegeneration pathways, and healthy aging.

## Results

### Expression levels of *SIRT1* and miRNAs in centenarian and AD LCLs

Human lymphoblastoid cell lines (LCLs) from healthy adults of various ages including non-demented centenarians and from AD patients were grown under optimal conditions in serum-containing medium. RNA samples were prepared from the 69 selected LCLs (see Methods).

**Figure 1:**
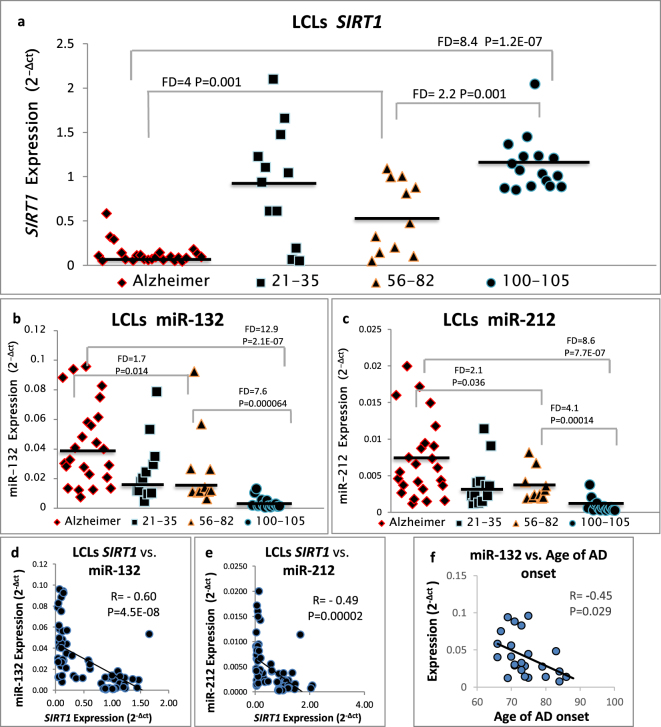
Expression levels and correlations of *SIRT1*, miR-132 and miR-212 in LCLs from female Alzheimer’s disease patients, female healthy controls (in two age groups) and female centenarians. Expression levels (2^−Δct^) are shown for the different LCL groups for: (**a**) *SIRT1* (**b**) miR-132 (**c**) miR-212. (**d**,**e**) Pearson correlation (R) plots for the expression levels of *SIRT1* with miR-132 and with miR-212 (combined for all cohorts; N = 69). The LCLs were from Alzheimer’s disease patients (N = 24), healthy controls aged 21–35 years (N = 12), healthy controls aged 56–82 years (N = 11) and centenarians (N = 16). (**f**) Pearson correlation between age of AD onset and miR-132 expression. Expression levels were determined by real-time PCR (see Methods). Bars represent the expression averages. Fold-difference (FD) values are shown between cohorts along with P values (Kruskal–Wallis test, Mann-Whitney U test). Average ages were similar for the AD patients (76 ± 1.4 years) and the 56–82 years control cohort (71 ± 2.7 years). Data for *SIRT1* expression levels in Alzheimer’s disease and control LCLs are from our previous study (Hadar *et al*. 2016), while new data were obtained for centenarian LCLs; miRNA expression levels were determined in the same RNA preparations (see Methods).

The expression levels of *RGS2* and *SIRT1*, both reported by us to be lower in AD vs. healthy LCLs^18^ were compared across the different age groups by real-time PCR. The healthy control LCLs were divided into three age groups: 21–35 years (mean 28 ± 1.4 years; N = 12), 56–82 years (mean 71 ± 2.6 years; N = 11), and non-demented centenarians aged 100–105 years (mean 101 ± 0.3 years; N = 16). Figure 1a depicts the individual and mean LCL expression levels of *SIRT1* across the non-demented age groups (N = 39) as well as the AD patients (N = 28). As shown, when compared to LCLs from individuals aged 56–82 years, the expression levels of *SIRT1* were upregulated (FD = 2.2; P = 0.001) in the centenarian LCLs. By contrast, *SIRT1* levels were similar in LCLs from centenarian donors compared with LCLs from individuals aged 21–35 years P = 0.45). In sharp contrast, the *SIRT1* expression levels in AD LCLs were dramatically down-regulated compared with centenarian LCLs (FD = −8.4; P = 1.2E-07) as well as compared with the LCLs from healthy donors aged 56–82 years (FD = −4.0; P = 0.001) or 21–35 years (FD = −6.8; P = 0.001; Fig. 1a).

The expression levels of both miR-132 and miR-212 showed a mirror-image of the *SIRT1* expression levels in the same LCL cohorts: levels were upregulated in AD LCLs compared with healthy age-matched control LCLs (FD = 1.7; P = 0.014, FD = 2.1; P = 0.036) and, more dramatically, were extremely low in centenarian compared with AD LCLs (FD = 12.9; P = 2.1E-07, FD = 8.6; P = 7.7E-07; Fig. 1b,c). These findings agree with the differential expression levels observed for *SIRT1* in the same RNA preparations. In addition, the expression levels of both miR-132 and miR-212 negatively correlated with those of *SIRT1* (R = −0.60 P = 4.5E-08; R = −0.49 P = 0.00002; Fig. 1d,e; data were combined for all cohorts, N = 69). Notably, miR-132 LCL expression levels, which negatively correlated with the respective *SIRT1* levels (Fig. 1d), negatively correlated also with AD age of onset (R = −0.45, P = 0.029) (Fig. 1f).

As shown in Supplementary Fig. 1a, miR-22 expression was down-regulated (FD = −2.5; P = 0.001) in the centenarian LCLs compared with controls aged 56–82 years. Negative Pearson correlation was observed between miR-22 and *SIRT1* expression levels (R = −0.629 P = 1.17E-09; Supplementary Fig. 1b). In an earlier study^18^ we reported lower *RGS2* expression in LCLs from AD patients compared with age-matched healthy controls. Here, we observed similar *RGS2* expression in the different healthy control age groups, including the centenarian LCLs (Supplementary Fig. 3b).

### AD LCLs expression levels of *SIRT1*, miR-132 and miR-212 correlate with patient cognitive scores

**Figure 2:**
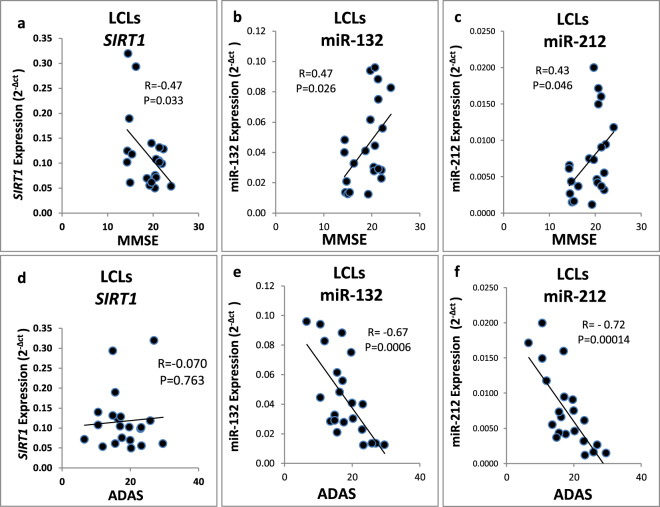
Pearson correlation plots for female Alzheimer’s disease MMSE and ADAS cognitive scores and expression levels (2^−Δct^) of *SIRT1*, miR-132 and miR-212 in their LCLs. (**a–c**) Pearson correlation plot for MMSE scores and expression levels of (**a**) *SIRT1* (N = 21*), (**b**) miR-132 (N = 22) and (**c**) miR-212 (N = 22). **(d**) Lack of correlation for ADAS scores and *SIRT1* expression levels (N = 21*). (**e**,**f**) Negative Pearson correlation plots for ADAS scores and expression levels of (**e**) miR -132 (N = 22) and (**f**) miR -212 (N = 22). ADAS and MMSE scores were recorded at time of blood withdrawal for LCL generation. Note the negative correlation of *SIRT1* expression with MMSE but not with ADAS; the negative MMSE correlation agrees with the positive MMSE correlations for miR-132 and miR-212, two miRNAs known to target *SIRT1* (see Discussion). ADAS, Alzheimer’s Disease Assessment Scale; MMSE, Mini-Mental State Examination. *A single outlier LCL was removed from the correlation analysis of ADAS and MMSE scores with *SIRT1*.

The expression levels of *SIRT1*, miR-132 and miR-212 in LCLs from female AD patients (N = 22) were examined for correlations with their Mini Mental State Examination (MMSE) scores and AD Assessment Scale (ADAS) scores (Supplementary Table 1). Higher ADAS scores and lower MMSE scores both indicate more severe cognitive deficits. *SIRT1* expression levels in AD LCLs showed negative correlation with patient MMSE scores (R = −0.47, P = 0.033) (Fig. 2a). Corresponding positive correlations were found when comparing MMSE scores and miR-132 (R = 0.47; P = 0.026) and miR-212 LCL expression (R = 0.43; P = 0.046; Fig. 2b,c). Patients ADAS score exhibited lack of correlation with *SIRT1* expression (Fig. 2d), albeit negative correlations were observed with expression levels of miR-132 (R = −0.67; P = 0.0006) or miR-212 (R = −0.72; P = 0.00014) (Fig. 2e,f). Of note, the LCL expression levels of miR-212 and miR-132 exhibited a robust positive Pearson correlation (R = 0.893; P = 6.12E-25; Supplementary Fig. 1c; combined cohorts, N = 69). This finding is not surprising given the proximal location of miR-212 and miR-132 on human chromosome 17p13.3 (separated by only 263 nucleotides) and was previously reported for chronic lymphocytic leukemia cells^41^.

### Expression levels of miR-132 and miR-212 are downregulated in AD postmortem olfactory bulb and hippocampus

**Figure 3:**
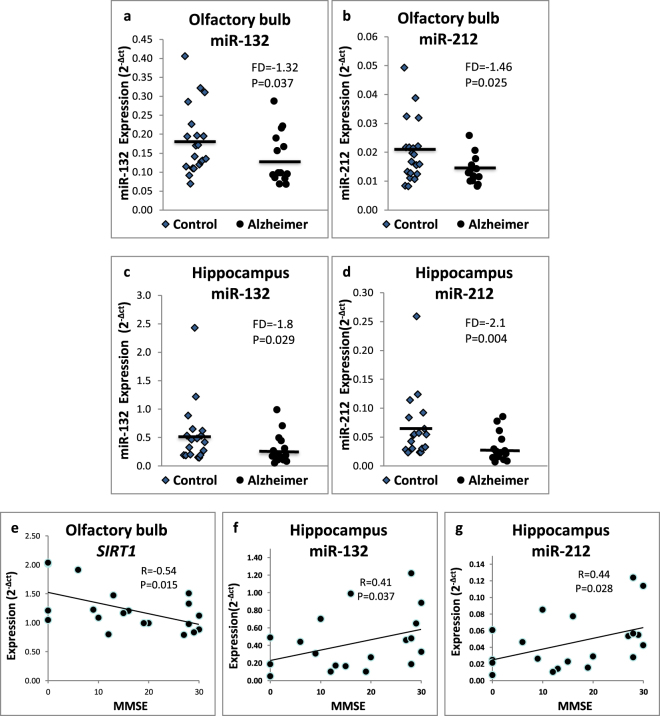
Expression levels of miR-132 (**a**,**c**) and miR-212 (**b**,**d**) in postmortem olfactory bulb (**a**,**b**) and hippocampus (**c**,**d**) tissues from sporadic Alzheimer’s disease patients and age-matched non-demented controls and their correlations with MMSE scores. Expression levels (2^−Δct^) were determined in postmortem tissue RNA preparations from Alzheimer’s disease patients (N = 14) and age matched non-demented controls (N = 20). See Methods for tissue collection and storage, RNA extractions, and real-time PCR experiments. Bars represent the miR expression averages. Fold-difference (FD) values are shown between cohorts along with P values (Mann-Whitney U test). (**e–g**) Pearson correlation between AD patients MMSE scores and their postmortem expression levels of (**e**) olfactory bulb *SIRT1*, (**f**) hippocampal miR-132 and (**g**) hippocampal miR-212.

We applied real-time PCR reactions for measuring the expression levels of *SIRT1*, *RGS2*, miR-22, miR-132 and miR-212 in postmortem olfactory bulb and hippocampus tissues from AD patients and non-demented age-matched controls. The expression levels of both miR-132 and miR-212 were downregulated in postmortem olfactory bulb (FD = −1.32; P = 0.037 and FD = −1.46; P = 0.025, respectively) and hippocampus tissues (FD = −1.8; P = 0.029 and FD = −2.1; P = 0.004, respectively) from late-onset AD patients (*N* = 14) compared to non-demented controls (*N* = 20) (Fig. 3a–d). However, similar *SIRT1* expression levels were observed in the same postmortem tissues from AD and non-demented controls. The expression levels of miR-212 and miR-132 exhibited negative Pearson correlations with *SIRT1* in the olfactory bulb tissues (Supplementary Fig. 4a,b). Additionally, *SIRT1* expression correlated with *RGS2* expression in the olfactory bulb tissues (Supplementary Fig. 4c). As observed in human LCLs, miR-132 expression levels correlated with those of miR-212 also in the olfactory bulb and hippocampus postmortem tissues (Supplementary Fig. 5a,b).

### *SIRT1*, miR-132 and miR-212 expression levels in the olfactory bulb and hippocampus are correlated with MMSE scores

The AD postmortem brain tissue MMSE cognitive scores were explored for detecting correlations with the measured expression levels of the above genes and miRNAs. A negative correlation was observed for the AD postmortem olfactory bulb expression levels of *SIRT1* with the patients MMSE cognitive scores (R = −0.54; P = 0.015); while in the hippocampus, both miR-132 and miR-212 showed positive correlations with MMSE scores (R = 0.41; P = 0.037, R = 0.44; P = 0.028; Fig. 3e–g). In addition, both miR-132 and miR-212 expression levels were negatively correlated with Braak stage scores and BrainNet Europe (BNE) Aβ phase score in AD olfactory bulb tissues (Supplementary Fig. 6).

## Discussion

In the current study, we compared the expression levels of *SIRT1* between LCLs derived from healthy controls of different age groups, including centenarians and AD patients. We observed upregulation of *SIRT1* expression in LCLs of centenarians compared with healthy controls aged 56–82 years (FD = 2.2, P = 0.01) and with AD patients (FD = 8.4, P = 1.2-E-07). Interestingly, *SIRT1* expression levels in LCLs of centenarians were similar to those of younger donors aged 21–35 (Fig. 1a).

Higher *SIRT1* levels afford axonal protection by increasing nicotinamide mononucleotide adenylyltransferase activity^42^. Mice overexpressing *Sirt1* exhibited repressed autoimmune encephalomyelitis clinical symptoms and reduced axonal injury^43^. SIRT1 protein was recently shown to deacetylate tau lysine residues in a mouse model of tauopathy, thereby reducing pathogenic tau generation and spread and suggesting that higher SIRT1 levels are neuroprotective^44^. SIRT1 protein levels were higher in blood of older healthy controls (56–92 years) compared with younger individuals (32–55 years)^45^. A study of *SIRT1* expression levels in PBMC reported lower expression in individuals aged 60–73 years or in nonagenarians and centenarians compared with younger individuals (19–42 years)^46^. However, the above studies included only samples from healthy controls. The purpose of our current study was to examine the expression levels of *SIRT1*, as well as miRNAs known to regulate it, in human LCLs and postmortem brain tissues from both AD patients and controls. One advantage of performing such studies in LCLs is that cell lines from all studied individuals are grown and analyzed under strictly similar conditions, thus minimizing variations arising from different methodological factors. A substantial part of the human genome is transcribed in both brain and LCLs, and indeed, these human cell lines have often proved instrumental for studying CNS disorder biomarkers^47^.

To further understand *SIRT1* regulation we tested (in the same LCL RNA preparations) the expression levels of miR-212^36^, miR-132^35^ and miR-22^37^ reported to regulate *SIRT1*, and observed opposite expression patterns compared with *SIRT1* (Fig. 1, Supplementary Fig. 1). We observed that miR-212 and miR-132 were upregulated in LCLs from AD patients compared to controls, and were extremely low in centenarian LCLs. These findings match the opposite expression patterns detected for *SIRT1* in the same cohorts suggesting that all three miRNAs take part in regulating *SIRT1* mRNA levels. In AD patients, lower *SIRT1* expression levels and higher miR-212 and miR-132 LCL expression levels correlated with better cognitive scores (higher MMSE and lower ADAS scores; Fig. 2), suggestive of earlier AD stage. In addition, higher miR-132 expression levels correlated with lower age of AD onset (Fig. 1f): the higher expression levels of miR-132 possibly allow improved defense against the ongoing neurodegeneration. The hypothesis that higher miR-212 and miR-132 levels are protective in AD shares common features with our recent suggestion that low peripheral *RGS2* expression may serve as an early AD biomarker, while lower *RGS2* expression levels were associated with better cognitive scores in AD patients^18^.

Indeed, higher serum miR-132 levels were recently suggested as biomarkers for mild cognitive impairment, a stage often preceding AD^48^. AD patients whose LCLs exhibited higher Aβ_1–42_ sensitivity had older age of disease onset (Supplementary Fig. 2c), while no age/Aβ_1–42_ sensitivity correlations were observed in healthy control LCLs (Supplementary Fig. 2a). Yet, no correlations were observed for *SIRT1* expression levels in LCLs with AD age of onset (Supplementary Fig. 2d). The expression levels of miR-212 and miR-132 in the hippocampus and olfactory bulb, two brain regions where neurogenesis takes place throughout human life, were downregulated in AD patient postmortem tissues compared with controls (Fig. 3a-d) and were correlated with worse cognitive MMSE scores (Fig. 3e–g). Our results confirm previous findings that miR-212 and miR-132 were downregulated in postmortem hippocampus brain tissues from AD patients^49^. Additionally, miR-132 and miR-212 were reduced in medial frontal gyrus^49^, temporal cortex, and gray matter from prefrontal cortex AD postmortem samples^50^. Nevertheless, our findings indicated similar *SIRT1* expression levels in postmortem olfactory bulb and hippocampus from AD patients compared with controls (Supplementary Fig. 7). *SIRT1* expression levels were decreased in miR-132 transfected human umbilical vein endothelial cells. These observations, which differ from those we observed in AD and control LCLs (Fig. 1 and Supplementary Fig. 3), may imply that the regulation of *SIRT1* expression by these miRNAs is tissue specific^51,52^.

miR-132 is required for normal dendrite maturation in newborn mouse hippocampal neurons^53^. miR-132 expression (measured by *in situ* hybridization) increases at the onset of synaptic integration in the mouse olfactory bulb. Furthermore sequestration of miR-132 in newborn neurons led to reduced dendritic complexity, spine density and increased survival of newborn neurons^54^. Additionally, mouse hippocampus miR-132 improves the integration of newborn neurons into synaptic circuitry^34^. miR-132 was involved in olfactory memory formation, as such, miR-132 expression was upregulated by odor exposure. Interestingly, a local infusion of miR-132 antisense into the olfactory bulb prior to training impaired olfactory learning in *C*. *sphinx*^55^.

Hypertension is recognized as an AD risk factor^56^. We recently proposed that the reduced *RGS2* expression we have identified during early AD progression^18^ increases angiotensin II receptor (AGTR2) signaling and thus contributes to hypertension, which may in part explain the comorbidity of hypertension and AD^57^. Interestingly, AGTR2 activation increases the expression of miR-212 and miR-132 in cardiovascular tissues^58^. Our current observations of higher miR-212 and miR-132 in AD LCLs may thus represent yet another facet of the hypertension/AD link.

**Figure 4:**
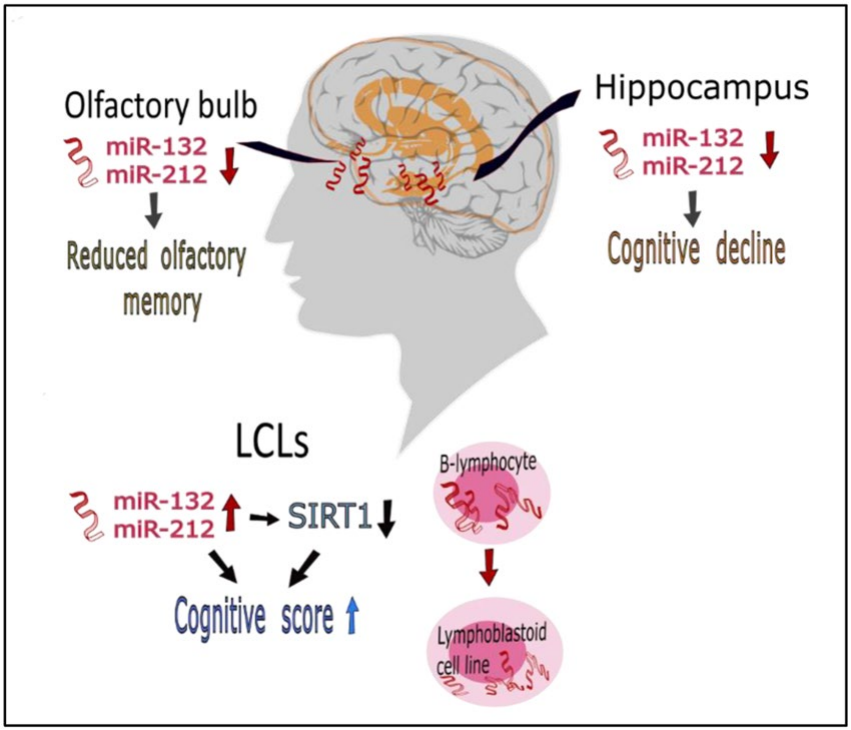
Scheme summarizing our key findings and hypotheses from AD LCLs and postmortem brain tissues, and proposing tentative disease-relevant events.

Our studies of miR-212 and miR-132 expression levels in AD and control LCLs yielded different findings compared with those obtained in postmortem brain tissues; this may reflect cross-talk between the immune system and the brain. The brain may reduce peripheral inflammation, possibly by parasympathetic cholinergic signaling which decreases cytokine production^59^. Transfection with miR-132 in normal B lymphocytes led to enhanced inflammation, as evidenced by increased production of lymphotoxin and tumor necrosis factor α^60^.Thus, the higher miR-132 expression we observed in AD LCLs (Fig. 1) is suggestive of an inflammatory-like state. Lipopolysaccharide-induced inflammation causes microglia activation near newborn neurons which impairs basal hippocampal neurogenesis in rats^61^. Additionally miR-132 was upregulated in U251 human astrocytoma cells exposed to the inflammatory protein myeloid related protein-8^62^. We propose that high levels of inflammation, known to take part in AD pathogenesis, may thus lead to higher levels of miR-132 in immune cells, as also reflected by AD LCLs in this study, allowing better cognitive status in AD patients through a pathway potentially involving *SIRT1* transcriptional down-regulation, as observed here (Fig. 4). Further studies are required for testing this hypothesis.

Our current findings highlight the key role of SIRT1 in healthy aging and in neurodegenerative disorders. *SIRT1* expression levels were much higher in centenarian compared with AD LCLs, while the opposite was observed for miR-132 and miR-212 in the same cells. Lower LCL miR-132 expression levels were associated with later AD age of onset. Furthermore, higher miR-132 and miR-212 levels were associated with improved AD patient cognitive scores. Thus, our observations in AD and centenarian LCLs further demonstrate their utility for studying CNS disorder biomarkers^47^. We suggest that miR-132 and miR-212 may take part in protection from neurodegeneration during early AD progression, a possibility that should be considered by further studies. In addition, our study highlights the value of including biosamples from centenarians in research on neurodegenerative diseases.

## Patients, Cell Lines, Materials and Methods

### Human LCLs

All LCLs were generated from peripheral blood B lymphocytes donated by consenting individuals. Of note, a written informed consent was obtained from all donors. LCLs from healthy adult donors aged 21 to 81 years were obtained from the National Laboratory for the Genetics of Israeli Populations (NLGIP; http://yoran.tau.ac.il/nlgip/) at Tel-Aviv University, Israel (N = 16) and from the University of Cagliari, Italy (N = 16). LCLs from AD patients were obtained from Prof. Alessio Squassina at the University of Cagliari (N = 28). LCLs from centenarians were obtained from Prof. Jacek Kuźnicki, the International Institute of Molecular and Cell Biology in Warsaw, Poland (N = 16; http://www.iimcb.gov.pl). Detailed demographic data of controls and cognitive scores of the AD patients are presented in Supplementary Table 1. Cells were maintained in similar optimal growth conditions as described^63^. Tissue-culture reagents were purchased from Biological Industries (Beit-Haemek, Israel).

### RNA extraction

RNA extractions were performed from cells incubated in upright T-25 flasks under optimal growth conditions in serum-containing media at a cell density of 0.5 × 10^6^-1 × 10^6^ cells/ml. Cells were centrifuged and then lysed using Tri-reagent (T9424, Sigma-Aldrich), followed by RNA separation using chloroform and precipitation using isopropanol and washed with 80% of cold ethanol. RNA was extracted using the phenol-chloroform method^63^. RNA was quantified using a NanoDrop spectrophotometer (NanoDrop, Wilmington, DE, USA), with both 260/280 nm and 260/230 nm parameters > 2.0.

### Postmortem AD and non-demented controls olfactory bulb and hippocampus

Forty postmortem olfactory bulb brain tissues and a similar number of hippocampus tissues (14 AD patients and 20 age matched non-demented controls) were obtained from Newcastle Brain Tissue Resource (NBTR) in accordance with the approval of the joint Ethics Committee of Newcastle and North Tyneside Health Authority and following NBTR brain banking procedures which includes full written informed consent from tissue donors or relatives. All AD cases fulfilled the criteria for high AD neuropathologic change according to the National Institute on Ageing-Alzheimer’s Association (NIA-AA) guidelines^64^. Demographic data for the AD patients and age matched non-demented controls are presented in Supplementary Table 2. RNAs were extracted from these postmortem tissues following homogenization using Bullet Blender (Next Advance, Inc., NY, USA), and extraction with ZR-Duet DNA-RNA MiniPrep Plus kit (Zymo Research, Irvine, CA, USA). Next, RNAs were quantified as described above for LCLs. Supplementary Fig. 8 describes our study design and includes N values for each cohort.

### Real-time PCR

Real-time quantitative PCR (qPCR) reactions were performed with cDNA samples prepared from 1 μg RNA samples using High Capacity cDNA Reverse Transcription kit (Applied Biosystems, Waltham, MA, USA) containing 10X RT buffer, 10X RT random primers, 25X dNTP mix, RNAse inhibitor and MultiScribe™ Reverse transcriptase. Reverse transcription was performed using a thermal cycler over three steps (25 °C for 10 min, followed by 37 °C for 120 min and 85 °C for 5 min). Real-time PCR reactions were done with 20 μl mixtures containing 20 ng of cDNA, Absolute Blue qPCR ROX mix (Thermo Scientific, MA, USA) and Primers (TaqMan® Gene Expression Assay (Applied Biosystems). *GUSB* (glucuronidase, beta) was used as reference gene as recommended for transcriptomic analysis of LCLs and for postmortem brain tissues^65^. TaqMan® Gene Expression Assay IDs are listed below:

**Table:** 

Gene Symbol	Assay ID
*GUSB*	Hs00939627_m1
*SIRT1*	Hs01009006_m1
*RGS2*	Hs01009070_g1

PCR reactions were performed using ABI Step One (Applied Biosystems) and the cycle protocol was as follows: 50 °C for 2 min, 95 °C for 15 min, followed by 40 cycles of 95 °C for 15 s and 60 °C for 1 min.

For measuring miRNA expression levels, cDNA samples were prepared from 350 ng RNA samples using High Capacity cDNA Reverse Transcription kit (Applied Biosystems, Waltham, MA, USA) in accordance with the protocol for Creating Custom RT Pools using TaqMan ® MicroRNA Assays from Applied Biosystems. Real-time PCR reactions were done with 10 μl mixtures containing 10 ng of cDNA, TaqMan® Fast Advanced Master Mix (Applied Biosystems, Waltham, MA, USA) and Primers (TaqMan™ MicroRNA Assay; Applied Biosystems). U6 snRNA was used as reference gene as recommended for transcriptomic analysis of LCLs^66^. TaqMan® MicroRNA Assay IDs are listed below:

**Table:** 

MicroRNA Symbol	TaqMan™ MicroRNA Assay ID
U6 snRNA (Control miRNA Assay)	001973
hsa-miR-22-3p	000398
hsa-miR-132-3p	000457
hsa-miR-212-3p	000515

Comparative critical threshold (Ct) values were determined in duplicates for analyzing relative gene and miRNA expression in selected sample groups according to 2^−ΔCт^ (ΔCт = Ct target Gene − Ct reference gene).

### Statistical analyses

Statistical analysis for gene and miRNA mean expression in LCLs and postmortem brain tissues was performed by Kruskal-Wallis test (for more than two groups) followed by non-parametric Mann-Whitney U test. Multiple testing corrections were performed by the Benjamini-Hochberg test^67^; adjusted P-values with a false discovery rate (FDR) < 0.05 were considered significant (Supplementary Table 3). Correlation analysis was performed by either Pearson correlation or Spearman correlation (as indicated in each relevant figure legend). P-values < 0.05 were considered significant. SPSS 23 software (SPSS Inc., Chicago, IL, USA) was used for all statistical analyses.

## Electronic supplementary material

Supplementary Figures
