# Supplementary material for: SIRT1, miR-132 and miR-212 link human longevity to Alzheimer’s Disease
Source: Sci Rep. 2018 May 31;8:8465. doi: 10.1038/s41598-018-26547-6 (PMC5981646; doi:10.1038/s41598-018-26547-6)
Supplement: Supplementary file 1 — Supplementary Figures [file 41598_2018_26547_MOESM1_ESM.pdf]

Supplementary materials for:

SIRT1, miR-132 and miR-212 link human longevity to Alzheimer's Disease

A Hadar, E Milanesi, M Walczak, M Puzianowska-Kuźnicka, J Kuźnicki, A Squassina, P Niola, C Chillotti, J Attems, I Gozes and D Gurwitz

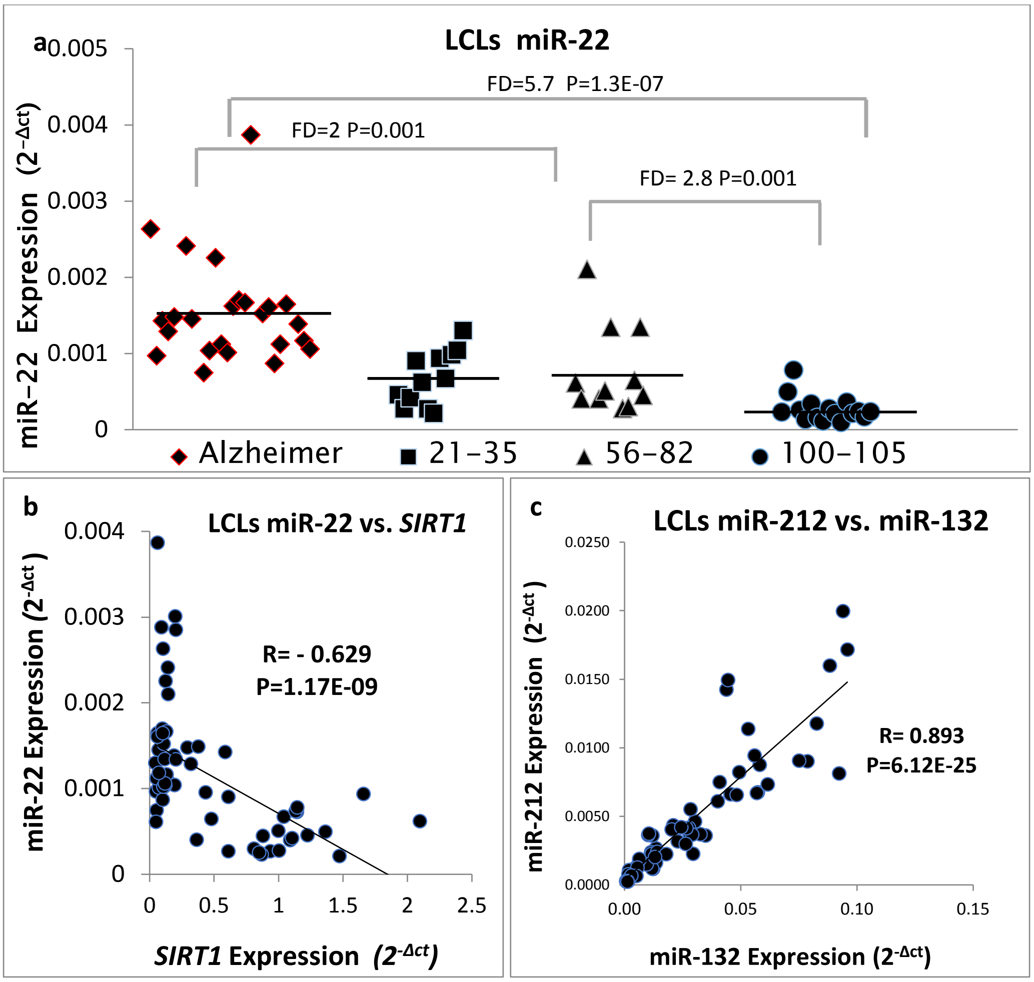

Supplementary Figure 1. miR-22 expression levels in human LCLs differ across age groups and show negative correlations with *SIRT1*. Expression levels ($2^{-\Delta ct}$) for miR-22, miR-212, miR-132, and *SIRT1* were measured in female LCLs from Alzheimer's disease patients (N=24), healthy controls aged 21-35 years (N=12), healthy controls aged 56-82 years (N=11), and centenarians (N=16): (**a**) miR-22 (**b**) Negative Pearson correlation between the expression levels of *SIRT1* and the expression levels of miR-22 in the combined LCL cohorts combined (N=69). (**c**) Pearson correlation between expression levels of miR-212 and miR-132 (N=69).

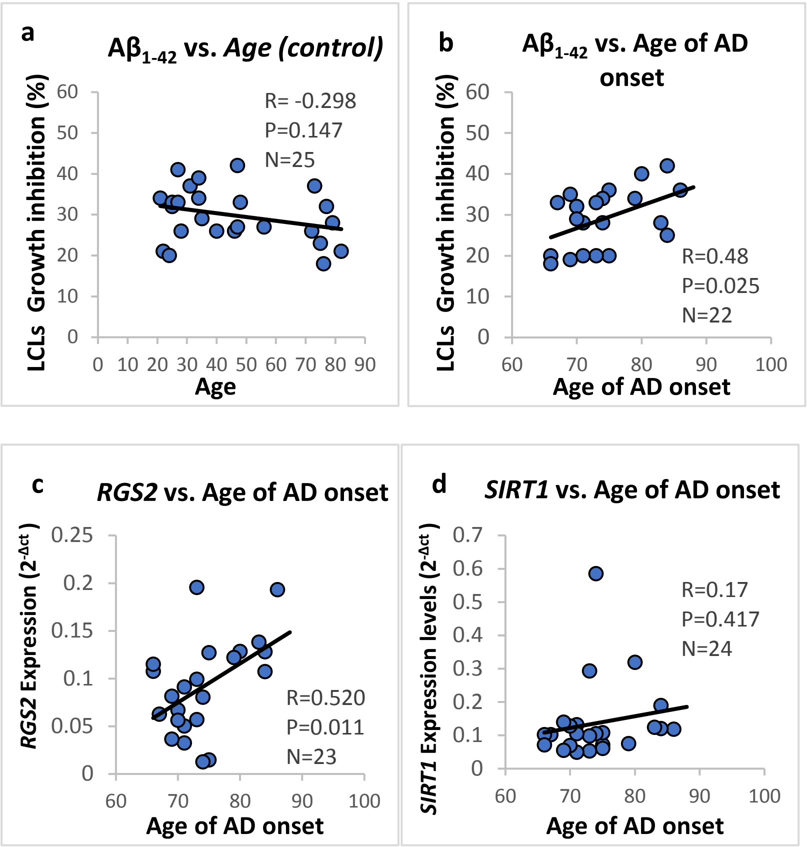

Supplementary Figure 2. Age of Alzheimer's disease (AD) onset and in vitro growth inhibition of LCLs by A$\beta_{1\text{-}42}$. (**a**) Lack of correlation between female control donors age and in vitro growth inhibition in their LCLs by 8 µM A$\beta_{1\text{-}42}$ (N=25). (**b-d**) Pearson correlations between age of disease onset in female AD patients and: (**b**) growth inhibition by 8 µM A$\beta_{1\text{-}42}$ in their LCLs (N=22). (**c**) *RGS2* expression levels in their LCLs (N=23). (**d**) *SIRT1* expression levels in their LCLs (N=24). Gene expression levels were determined by real-time PCR (see Methods); growth inhibition by A$\beta_{1\text{-}42}$ was determined by the XTT cell proliferation kit (see Supplementary Methods).

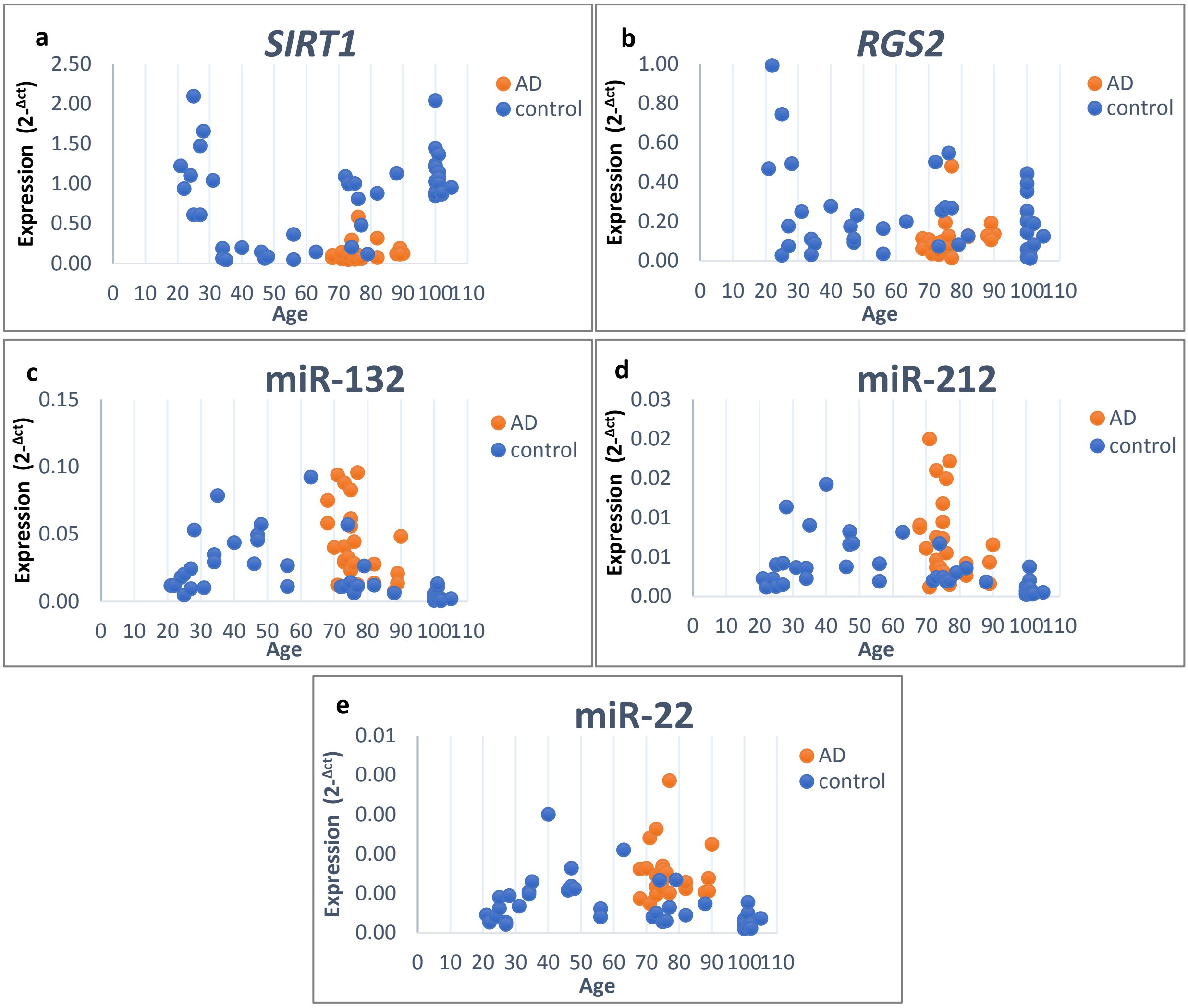

Supplementary Figure 3. Scatter plots of *SIRT1, RGS2*, miR-132, miR-212 and miR-22 expression levels in female LCLs across their age. Gene expression levels were determined by real-time PCR (N=69; see Methods).

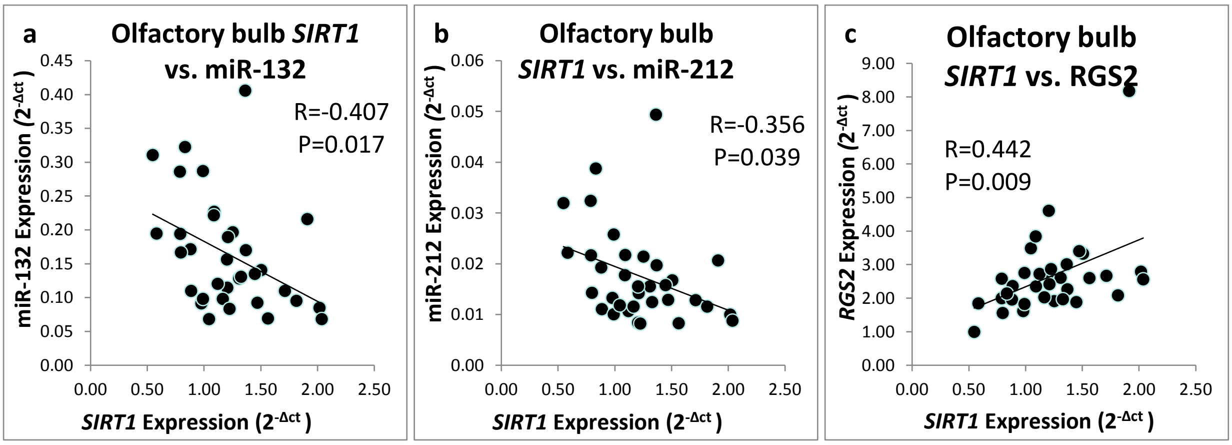

Supplementary Figure 4. Pearson correlations between the expression levels of studied genes and miRNAs in human olfactory bulb postmortem tissues. Data are from real-time PCR experiments in the combined olfactory bulb postmortem tissues from AD patients and age-matched non-demented controls (N=34; see Methods).

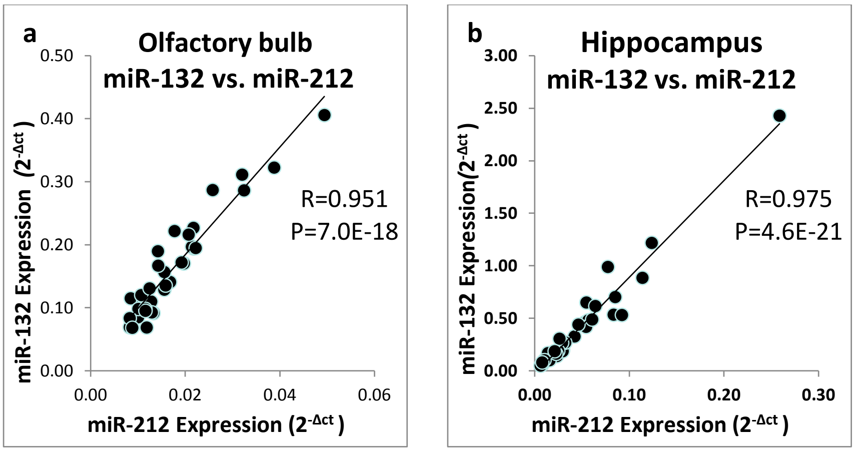

Supplementary Figure 5. Pearson correlations between the expression levels of miR-212 and miR-132. (**a**) olfactory bulb (N=34). (**b**) Hippocampus (N=32). Data are from real-time PCR experiments in the combined cohorts of olfactory bulb and hippocampus postmortem tissues from AD patients and age-matched non-demented controls (see Methods).

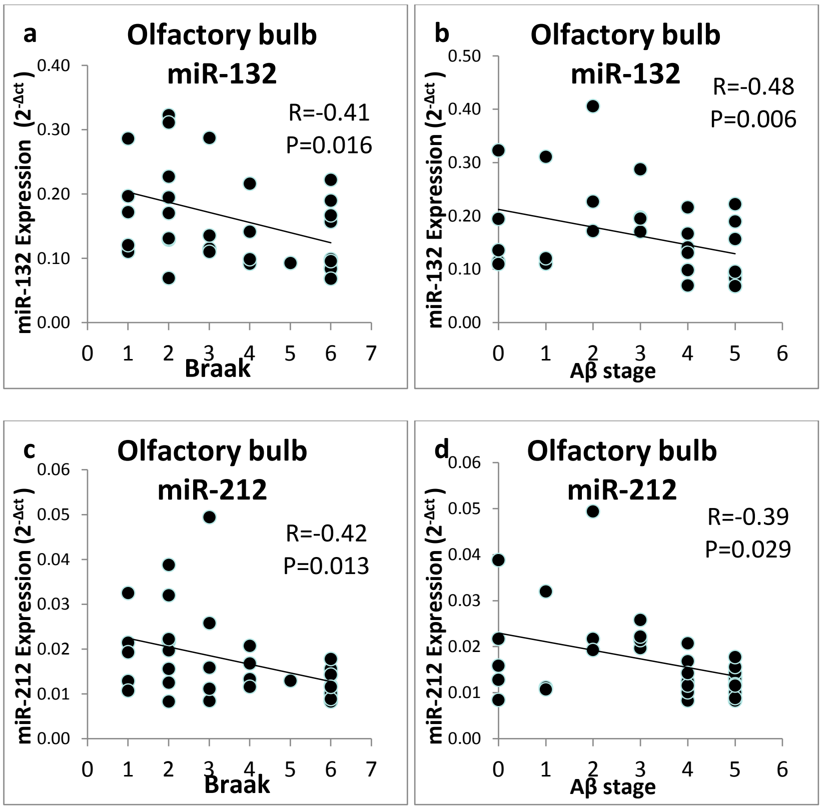

Supplementary Figure 6. Spearman correlations between the expression levels of miR-132 or miR-212 and the postmortem olfactory bulb Braak and Aβ stages of the combined AD patients and age-matched controls. (**a,c**) Correlations with Braak stages (N=34). (**b,d**) Correlations with Aβ stages (N=32).

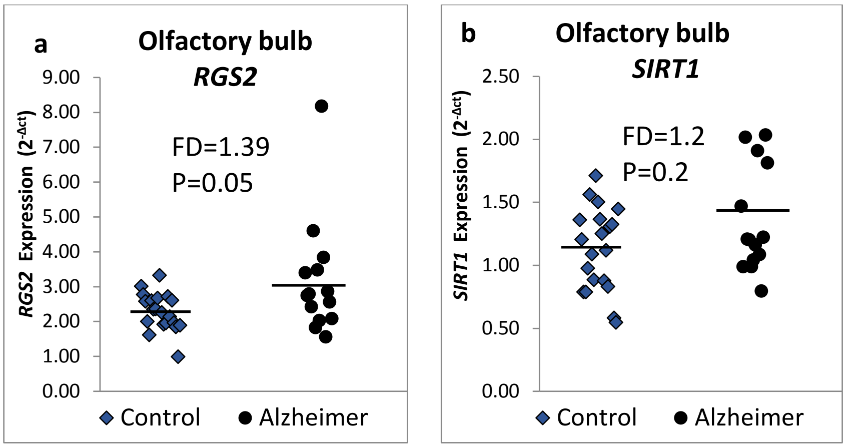

Supplementary Figure 7. Postmortem olfactory bulb expression levels. Expression levels ($2^{-\Delta ct}$) are compared for (**a**) *RGS2* and (**b**) *SIRT1* in AD patients (N=14) and age-matched non-demented controls (N=20).

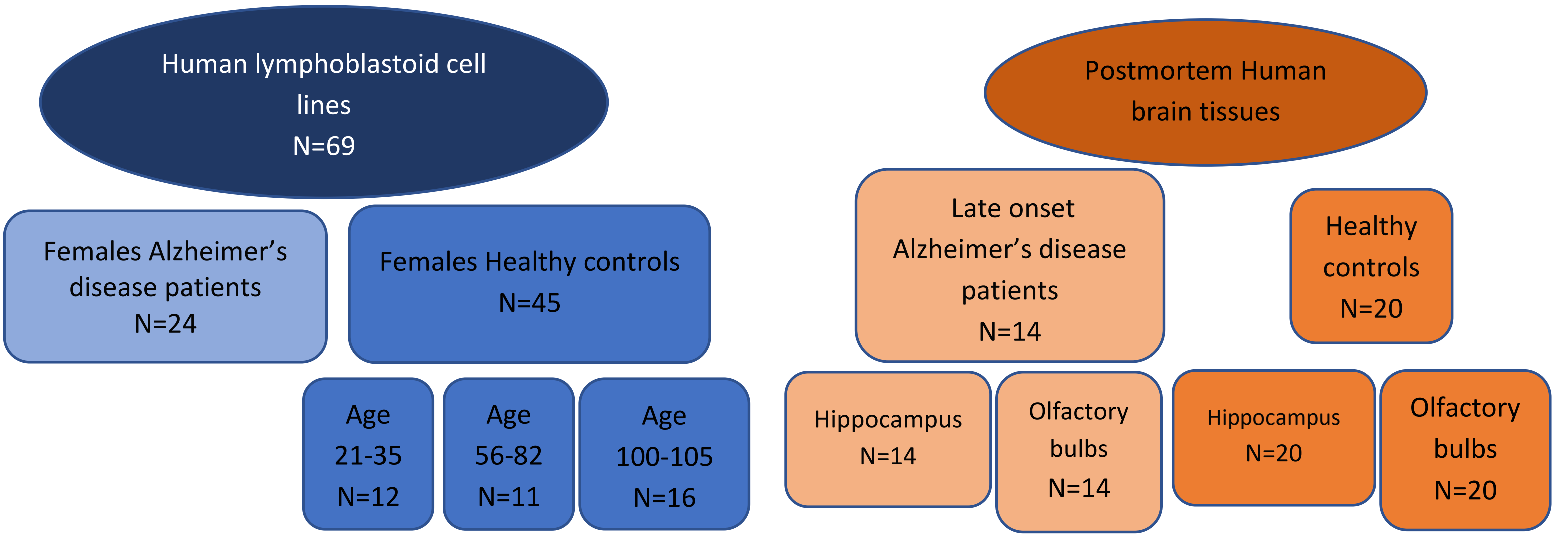

Supplementary Figure 8. A flowchart presenting the study design.

Supplementary Table 1: (a) Demographic data for Alzheimer's disease patients (LCLs cohort). Sex, age, age at first AD diagnosis, MMSE and ADAS scores (at time of blood collection for LCL generation) are shown for 24 female AD patients.

ID sample	*Sex*	*Age at sampling (years)*	*Age at onset (years)*	*MMSE*	*ADAS*
**1162**	F	90	83	14.4	16.3
**1088**	F	89	86	15.4	25.9
**1092**	F	89	84	14.8	15.6
**1170**	F	88	84	NA	NA
**1150**	F	82	79	20.5	17.6
**1215**	F	82	80	14.5	27
**1132**	F	77	75	15	29.6
**1149**	F	77	75	20.7	6.6
**1130**	F	76	75	20.7	10.6
**1219**	F	76	74	22	13.6
**1110**	F	75	73	24	11.9
**1121**	F	75	74	19.7	15.6
**1135**	F	75	70	22.3	17.2
**1214**	F	74	73	16.3	14.9
**1090**	F	73	71	21.4	14.9
**1187**	F	73	70	18.7	20
**1229**	F	73	71	20.4	20.3
**1235**	F	73	71	21.3	17
**1173**	F	71	69	19.3	23.3
**1197**	F	71	69	19.7	10.6
**1100**	F	70	66	14.3	23.2
**1118**	F	68	67	21.4	19.7
**1141**	F	75	73	22	23
**1145**	F	68	66	NA	NA

(b) Demographic data for healthy adult controls (LCLs cohort).

ID sample	***Sex***	***Age (years)***
**1012**	F	56
**1016**	F	47
**1060**	F	79
**1376**	F	56
**1379**	F	74
**1446**	F	63
**1466**	F	34
**1706**	F	35
**1728**	F	48
**1744**	F	46
**1791**	F	47
**1793**	F	40
**1799**	F	34
**1728**	F	22
**1516**	F	75
**1370**	F	24
**1515**	F	88
**1389**	F	28
**1978**	F	76
**1518**	F	82

ID sample	***Sex***	***Age (years)***
1130	F	72
1146	F	21
6037	F	77
1826	F	27
1823	F	25
1549	F	73
1754	F	25
1801	F	27
1128	F	31
216	F	100
301	F	100
343	F	100
349	F	100
352	F	100
353	F	100
446	F	100
450	F	100
176	F	102
346	F	102
361	F	105

ID sample	***Sex***	***Age (years)***
574	F	100
180	F	101
197	F	101
298	F	101
516	F	101

Supplementary Table 2: Demographic data for Alzheimer's disease patients and healthy controls (cohort of postmortem brain tissues). Sex, average ages and last MMSE scores are shown.

Group	N	Male	Female	Age	MMSE
**Alzheimer's disease**	14	10	4	88.2±6.2	9.2±7.5
**Control**	20	11	9	86.5±10.5	28.5±1.1

Supplementary Table 3: Benjamini-Hochberg adjusted P-values for multiple testing. Adjusted p-values with FDR< 0.05 (in bold fonts) were considered significant. Statistical analyses are described under Methods. Two-tailed non-parametric Mann-Whitney U test was applied for all genes and miRNAs tested; while one-tailed Mann-Whitney U test was applied for miR-132 and miR-212 in postmortem brain tissues (as previous findings indicated that these two miRNAs were downregulated in postmortem hippocampus brain tissues from AD patients[48]).

LCLs Alzheimer vs 100-105 group	P-value (two-tailed Mann-Whitney U test	Benjamini-Hochberg adjusted P-value
SIRT1	**.00000012**	**.00000034**
miR-22	**.00000013**	**.00000034**
**miR-132**	**.00000021**	**.00000035**
miR-212	**.00000077**	**.00000097**
RGS2	.3203	.3203

Alzheimer vs 56-82 group	P-value (two-tailed Mann-Whitney U test	Benjamini-Hochberg adjusted P-value
miR-22	**.00108**	**.00391**
SIRT1	**.00156**	**.00391**
**miR-132**	**.01421**	**.01960**
RGS2	**.01568**	**.01960**
miR-212	**.03604**	**.03604**

56-82 group vs 100-105 group	P-value (two-tailed Mann-Whitney U test	Benjamini-Hochberg adjusted P-value
miR-132	**.000064**	**.000321**
miR-212	**.000145**	**.000362**
**miR-22**	**.000552**	**.000920**
SIRT1	**.001339**	**.001674**
RGS2	.300	.300

**Hippocampus Alzheimer vs control**	**P-value (one-tailed Mann-Whitney U test**	**Benjamini-Hochberg adjusted P-value**
**miR-212**	**.0040**	**.0080**
**miR-132**	**.0290**	**.0290**
**Olfactory bulb Alzheimer vs control**	**P-value (one-tailed Mann-Whitney U test**	**Benjamini-Hochberg adjusted P-value**
**miR-212**	**.0250**	**.0370**
**miR-132**	**.0370**	**.0370**

**Hippocampus Alzheimer vs control**	**P-value (two-tailed Mann-Whitney U test**	**Benjamini-Hochberg adjusted P-value**
SIRT1	.402	.849
RGS2	.827	.849
miR-22	.849	.849
**Olfactory bulb Alzheimer vs control**	**P-value (two-tailed Mann-Whitney U test**	**Benjamini-Hochberg adjusted P-value**
RGS2	.0500	.1501
SIRT1	.2207	.3310
miR-22	.4624	.4624

Supplementary Methods

Cell proliferation assay

Cell growth inhibition was examined in vitro in LCLs by exposure to 8 μM pre-aggregated $A\beta_{1-42}$ fibrils for 3 days, as described[18]. LCLs were first washed in PBS and suspended with serum-free RPMI medium containing 4% BIOGRO-2 (Biological Industries, Beit-Haemek, Israel). This BIOGRO-2 concentration was optimal for long-term serum-free growth of LCLs[1]. The serum-free conditions are essential for observing $A\beta_{1-42}$ mediated growth inhibition. Cells were counted and diluted in the same media to a concentration of 250,000 cells/ml, followed by plating 100 μl per well in 96-well plates (Corning Incorporated, NY, USA). Amyloid-β_{1-42} ($A\beta_{1-42}$) peptide was purchased from Genemed Synthesis Inc. (San Antonio, TX, USA). $A\beta_{1-42}$ peptide was dissolved in sterile tissue-culture grade water (1 mg/ml) and stored (as 100 μl aliquots) at -20°C. Prior to experiments, an aliquot of $A\beta_{1-42}$ in water was pre-incubated at 37 °C for 3 days[2,3] for assuring the generation of Aβ fibrils[4,5]. Following $A\beta_{1-42}$ treatment, LCL proliferation was assayed by the XTT cell proliferation kit (Biological Industries, Israel) as described[6]. Each cell line was tested for of its sensitivity to $A\beta_{1-42}$ (in triplicate) in three independent experiments.

References for Supplementary Methods*

1. Milanesi E, Hadar A, Maffioletti E, et al. Insulin-like Growth Factor 1 Differentially Affects Lithium Sensitivity of Lymphoblastoid Cell Lines from Lithium Responder and Non-responder Bipolar Disorder Patients. *Journal of molecular neuroscience : MN.* 2015;56(3):681-687.
2. Morishima Y, Gotoh Y, Zieg J, et al. Beta-amyloid induces neuronal apoptosis via a mechanism that involves the c-Jun N-terminal kinase pathway and the induction of Fas ligand. *The Journal of neuroscience : the official journal of the Society for Neuroscience.* 2001;21(19):7551-7560.
3. Elkind E, Vaisid T, Kornspan JD, Barnoy S, Rottem S, Kosower NS. Neuroprotective effects of Mycoplasma hyorhinis against amyloid-beta-peptide toxicity in SH-SY5Y human neuroblastoma cells are mediated by calpastatin upregulation in the mycoplasma-infected cells. *Neurochemistry international.* 2011;58(4):497-503.
4. Soreghan B, Kosmoski J, Glabe C. Surfactant properties of Alzheimer's A beta peptides and the mechanism of amyloid aggregation. *The Journal of biological chemistry.* 1994;269(46):28551-28554.
5. Bernstein SL, Wyttenbach T, Baumketner A, et al. Amyloid beta-protein: monomer structure and early aggregation states of Abeta42 and its Pro19 alloform. *Journal of the American Chemical Society.* 2005;127(7):2075-2084.
6. Morag A, Pasmanik-Chor M, Oron-Karni V, Rehavi M, Stingl JC, Gurwitz D. Genome-wide expression profiling of human lymphoblastoid cell lines identifies CHL1 as a putative SSRI antidepressant response biomarker. *Pharmacogenomics.* 2011;12(2):171-184.

*This file also cites articles listed in the main article file.
